# A Pilot Project of Early Integrated Traumatic Brain Injury Rehabilitation in Singapore

**DOI:** 10.1155/2014/950183

**Published:** 2014-05-21

**Authors:** Siew Kwaon Lui, Yee Sien Ng, Annie Jane Nalanga, Yeow Leng Tan, Chek Wai Bok

**Affiliations:** ^1^Department of Rehabilitation Medicine, Singapore General Hospital, 20 College Road, Academia Level 4, Singapore 169856; ^2^Duke-National University of Singapore (NUS) Graduate Medical School, 8 College Road, Singapore 169857

## Abstract

*Objective.* Document acute neurosurgical and rehabilitation parameters of patients of all traumatic brain injury (TBI) severities and determine whether early screening along with very early integrated TBI rehabilitation changes functional outcomes. *Methods*. Prospective study involving all patients with TBI admitted to a neurosurgical department of a tertiary hospital. They were assessed within 72 hours of admission by the rehabilitation team and received twice weekly rehabilitation reviews. Patients with further rehabilitation needs were then transferred to the attached acute inpatient TBI rehabilitation unit (TREATS) and their functional outcomes were compared against a historical group of patients. Demographic variables, acute neurosurgical characteristics, medical complications, and rehabilitation outcomes were recorded. *Results.* There were 298 patients screened with an average age of 61.8 ± 19.1 years. The most common etiology was falls (77.5%). Most patients were discharged home directly (67.4%) and 22.8% of patients were in TREATS. The TREATS group functionally improved (*P* < 0.001). Regression analysis showed by the intervention of TREATS, that there was a statistically significant FIM functional gain of 18.445 points (95% CI −30.388 to −0.6502, *P* = 0.03). *Conclusion.* Our study demonstrated important epidemiological data on an unselected cohort of patients with TBI in Singapore and functional improvement in patients who further received inpatient rehabilitation.

## 1. Introduction

Traumatic brain injury (TBI) is a significant medical, social, and public healthcare problem worldwide [1]. More than 1.7 million people sustain TBI annually in the United States [2].

Trauma was the fifth leading cause of death locally in 2011, and it was the leading cause of deaths in persons under 45 years which contributed more than a third of deaths in this group [3].

Traumatic brain injury results in high mortality and morbidity with large numbers of patients sustaining permanent disability [4].

The economic cost of TBI is tremendous with the annual economic burden of TBI in the United States approximated at US $76.5 billion [5, 6]. A local study of 91 TBI selected admissions to rehabilitation over a 2-year period indicates a total median rehabilitation charge per episode [7] of S $7845.50.

Although health-care costs associated with TBI are substantial, studies have shown that rehabilitation of TBI patients is cost-effective [8] with reduction of mortality by approximately 3607 lives annually [9].

Very early rehabilitation is characterized by rehabilitation in acute medical units commencing as soon as patients are medically stable. In a very early rehabilitation trial for stroke (AVERT), it seems to be safe to start ambulatory therapy in patients who satisfied physiologic safety criteria within 24 hours of stroke [10]. This optimizes early neuroplastic changes leading to better recovery [11–13]. Very early rehabilitation also has benefits of faster improvement in independence and better reported quality of life [14, 15].

Although there are benefits associated with very early rehabilitation, there is often a paucity of a coordinated effort to address the rehabilitation needs early in the acute medical units where the primary focus is to manage the acute medical problems.

There are datasets of TBI demographics in several national databases, but most of the patient cohorts are generally of moderate or severe TBI which do not represent the entire TBI spectrum [16–18]. There are also acute neurosurgical TBI databases, but these mainly contain acute surgical data and provide limited information on rehabilitation characteristics and functional outcomes [19].

Hence the objectives of the study are as follows:

- document both acute neurosurgical clinical characteristics and rehabilitation data of patients of all TBI severities admitted into the acute hospital;
- determine whether early screening and provision of very early integrated TBI rehabilitation service changes functional outcomes in the group who received further inpatient rehabilitation.

## 2. Methods

### 2.1. Study Design, Setting, and Participants

This prospective study involved patients of all TBI severities presenting through Department of Neurosurgery (NES) of Singapore General Hospital (SGH) between November 1, 2010, and February 15, 2012.

The patients were screened within 72 hours of admission. Patients were included in our study if they had a diagnosis of TBI. The diagnosis of TBI was made through an appropriate clinical history and examination by the admitting team and supported with computed tomography or magnetic resonance imaging brain scans.

### 2.2. Very Early Integrated TBI Rehabilitation

All patients with TBI received regular twice weekly multidisciplinary reviews from the physiatrist-led rehabilitation team. This team included a nurse, physiotherapist, occupational therapist, speech therapist, dietician, and medical social worker. The aims of the review included verifying data accuracy and formulating rehabilitation plans. Our definition of very early integrated TBI rehabilitation consisted of TBI rehabilitation, while the patients were still in the acute NES unit and it served as a coordinated effort by the rehabilitation team to manage the rehabilitation issues early and facilitate functional recovery. Depending on individual needs and the medical condition of the patient, patients received approximately half an hour to two hours of therapy per day, 5 days of the week. In consultation with the multidisciplinary team, all patients received an individual specific discharge plan. Patients who were medically stable and required further inpatient rehabilitation were then further transferred to the acute inpatient rehabilitation unit of the Department of Rehabilitation Medicine, SGH, or to subacute rehabilitation facilities at the local community hospitals. The need for further inpatient rehabilitation was determined by the physiatrist-led team at the twice weekly reviews and examples of such needs included management of disorders of consciousness, motor, sensory, and cognitive deficits, language impairments, and neurobehavioral problems. The parameters collected were determined based on (1) the best available literature on known factors predicting recovery after TBI, (2) country, social, and cultural specific data, and (3) multidisciplinary team consensus meetings.

### 2.3. Clinical Variables and Outcome Measures

The data of the acute admissions with TBI were categorized into the following categories:

1. demographic variables including age, gender, and race;
2. acute neurosurgical characteristics including etiology, severity measured via Glasgow Coma Scale (GCS) upon admission, neuroimaging findings, and neurosurgical interventions;
3. comorbidities and complications including nosocomial infections and need for tracheostomy;
4. acute inpatient stay data including the acute length of stay (ALOS) and the discharge disposition;
5. rehabilitation outcomes including the Functional Independence Measure (FIM), Ranchos Los Amigos Score (RLA), and the Westmead Post-Traumatic Amnesia Score (PTA).

In order to evaluate whether early screening and provision of very early integrated TBI rehabilitation service changes functional outcomes, we compared functional outcomes of the TREATS group of patients against the historical group.

The definition of the TREATS group was patients with TBI who underwent early screening and received further inpatient rehabilitation at the SGH Department of Rehabilitation Medicine.

The definition of the historical group was patients with TBI who received inpatient rehabilitation in the same acute rehabilitation unit prior to the implementation of the early “reach-in” screening program and these patients were usually referred to by the primary department, that is, Department of NES. Data of the historical group of patients were obtained from the Rehabilitation Database of the Department of Rehabilitation Medicine, SGH.

The difference in outcomes between the TREATS and the historical groups was measured by comparison of the FIM gain, the acute length of stay (ALOS), and the rehabilitation length of stay (RLOS) of the 2 groups.

Patients aged 65 or more were defined as geriatric patients [20].

The etiologies of TBI were listed as falls, road traffic accidents, sports, assault, and others. For the patients who fell, the mechanisms of falls were further classified into mechanical falls (e.g., slipped and fell on wet surface), nonmechanical falls (e.g., fell as a result of muscle weakness), and unwitnessed falls. This distinction is important as patients will require further investigations for falls in the presence of medical stressors [21]. The GCS was documented on admission and used to classify the severity of TBI into mild (GCS 13–15), moderate (9–12), and severe (GCS 3–8) categories.

The main types of TBI were based on predominant neuroimaging findings. These were categorized into subdural hematoma, subarachnoid hemorrhage, contusion, intracerebral hemorrhage, and extradural hemorrhage. Patients were defined as having a concussion if there were no structural abnormalities on neuroimaging but with presence of “physical, cognitive, emotional, and/or sleep-related symptoms that may or may not involve a loss of consciousness” [22]. All neurosurgical interventions were recorded. Comorbidities and complications such as nosocomial infections, deep venous thrombosis, and seizures were documented.

The ALOS was recorded for all patients who were acutely screened and was defined as the time from admission to the hospital to discharge to home, an acute/subacute rehabilitation unit or a nursing home. The RLOS was defined as the time from admission to the SGH inpatient rehabilitation unit to discharge to home, subacute rehabilitation facility, or a nursing home.

The main functional outcome measure is the Functional Independence Measure (FIM) which is a widely used standardized functional outcome measure in medical rehabilitation [23]. It consists of 13 motor and 5 cognitive items, with established content and construct validity, sensitivity, and interrater reliability for the measurement of general functional ability across a wide range of rehabilitation conditions. Scores range from 1 (totally dependent) to 7 (totally independent) for each of the 18 items, with a maximum score of 126 indicating total functional independence. The FIM was recorded during the first assessment of all the patients within 72 hours of admission.

The FIM gain which is the difference between rehabilitation discharge and admission FIM were recorded for the TREATS and historical groups as it is a measure of functional improvement. The FIM gain of the 2 groups was compared against one another to determine whether functional outcomes were different between the 2 groups.

Posttraumatic amnesia (PTA) was documented as it is a strong predictor of recovery after TBI. The Westmead PTA Scale is used in our study to determine the severity of memory and cognitive impairment in TBI in addition to the commonly used GCS [24, 25]. The PTA scale was administered when patients were alert and able to communicate intelligibly. Daily scores were obtained by the rehabilitation team until the patient emerged from PTA or was discharged from the acute admission. The total duration of PTA included the time from TBI to the first day the patient achieved 3 consecutive full scores of 12/12 prior to discharge.

The Rancho Los Amigos Levels of Cognitive Function Scale (RLA) is used to assess cognitive functioning in postcoma patients for the planning of treatment, tracking of recovery, and classifying outcome levels [26, 27]. This scale comprises of levels from I to VIII. The RLA scale was recorded for patients who were out of general sedation upon first assessment.

Further data on patient stay in the subacute rehabilitation facilities at the community hospitals were unobtainable.

### 2.4. Statistical Analysis

Data was recorded on Microsoft Excel 97–2003 and was analyzed with SPSS Statistics for Windows, version 17.0. Chicago: SPSS Inc. We conducted descriptive analyses on the demographics of the patients who were acutely screened, their acute neurosurgical characteristics, comorbidities and complications, and rehabilitation outcomes.

Spearman's rank correlation coefficient was used to evaluate the association between ALOS and variables such as age, GCS, and total FIM in the total cohort of screened patients with TBI.

Differences between groups such as men and women in the total cohort of patients with TBI and between the TREATS and historical subgroups were analyzed using nonparametric tests when they were not of normal distributions.

Regression analysis was carried out to evaluate for clinical variables associated with FIM gain in the TREATS group.

A *P* value less than 0.05 was considered to be statistically significant.

This study was approved by the hospital institutional review board.

## 3. Results

### 3.1. Demographics, Acute Neurosurgical, and Rehabilitation Data

There were 298 patients with TBI during the study period from November 1, 2010, and February 15, 2012.

The average age of the cohort was 61.8 ± 19.1 years (range 15–99) with a significant difference of the women being older than the men in the cohort (*P* < 0.001) (Table 1). Almost half of the cohort (49.0%) was geriatric patients (Table 2). There were almost twice as many males who sustained a TBI (male/female ratio 1.9 : 1).

The most common etiology of the TBI was falls (77.5%), followed by road traffic accidents (10.0%) (Table 1). Less common etiologies were assault and sports. There was a statistically significant difference between genders when compared for the etiologies of TBI (*P* = 0.03) (Table 1).

Within the fall category, 61.9% were males and almost half (48.7%) of the falls were of nonmechanical origin. These included patients who fell due to syncope or other neurological symptoms. Thirty one percent of falls were mechanical in origin, while unwitnessed suspected falls constituted 20.2% of the fall cohort. Among those who fell, 57.8% were aged 65 and above (*P* = 0.018) and the most number of falls happened in the age group 70–79 years (Table 2) with a high significance of falls in the geriatric age group (91.8% versus 63.8%, *P* < 0.001).

**Figure 1:**
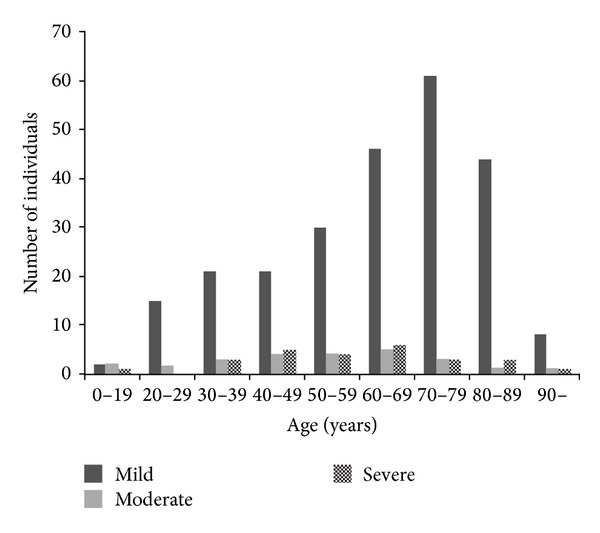
Distribution of severity of brain injury according to age groups.

The admission GCS scores indicated that the majority of patients (83.2%) sustained mild TBI (Table 1) and there seemed to be an increase in frequency of mild TBI with increasing age with the highest number in patients aged 70–79 years old (Figure 1). There was no significant difference between the 195 men and 103 women regarding severity of injury based on GCS scores on admission (*P* = 0.37) (Table 1). The majority of patients (67.3%) sustained subdural hematoma (SDH) or subarachnoid hemorrhage (SAH) or both SDH and SAH (Table 3).

Sixty-two patients (20.8%) received various neurosurgical interventions such as burr hole drainage, craniectomy, and external ventricular drain insertion. Six patients (2%) required tracheostomy.

Forty-eight patients (16.1%) had nosocomial infections with the 2 most common infections being pneumonia and urinary tract infection (Table 3).

About half of the TBI patients (50.7%) were in the RLA VIII category (Table 4).

More than half of the TBI patients (63.8%) were in PTA during assessment.

Most of the patients (67.4%) from the acute neurosurgical ward were discharged home. About a quarter of the patients (25.8%) required further inpatient rehabilitation in a rehabilitation facility and majority of these patients (88.3%) were transferred to the Department of Rehabilitation Medicine of SGH (TREATS). The remaining 9 patients were transferred to subacute rehabilitation facilities at the community hospitals. There was a statistically significant difference between the TREATS group of patients and the directly discharged group of patients in the admission GCS scores (13 versus 14, resp.; *P* < 0.001) and admission total FIM (51.1 versus 85.7, resp.; *P* < 0.001). Seven (2.3%) patients were discharged to a nursing home facility. Eight men (mean age 61.6, range 38–84) and 5 women (mean age 74.8, range 54–97) died. Out of these 13 patients, 5 sustained a severe TBI, 1 a moderate TBI, and 7 a mild TBI.

The mean total FIM of the 298 patients on admission was 71.6 ± 40.0 (range 18–126). The median FIM score was 76.5. The mean motor FIM score was 48.3 ± 29.7 (range 13–91) and the mean cognitive FIM score was 23.3 ± 12.2 (range 5–35). There was no significant difference in the FIM scores on admission between men and women (Table 1).

The average ALOS in the cohort of 298 patients with TBI was 19.9 ± 28.7 days (range 1–199). The ALOS was shown to have a moderately negative correlation with GCS on admission (*r* = −0.387, *P* < 0.001) and total FIM (*r* = −0.517, *P* < 0.001). However, the relationship between ALOS and age was poor (*r* = −0.140, *P* = 0.015).

### 3.2. Outcome and Subgroup Analyses of TREATS Group

Sixty-eight patients were in the TREATS group. This was compared against a group of 51 historical patients.

The mean age of TREATS was 66.2 ± 17.0 (range 15–92), whereas the mean age of the historicals was 47.8 ± 20.1 (range 14–81) (*P* < 0.001).

Patients in the TREATS group showed functional improvement during the inpatient rehabilitation stay as demonstrated by statistically significant changes in FIM (*P* < 0.001) (Table 5).

There was no statistically significant difference between the TREATS and historical groups regarding the total FIM on admission and upon discharge (Table 5). There was also no difference in the total FIM gain, motor FIM gain, and cognitive FIM gain in both groups (Table 5).

The total LOS of TREATS was longer than historicals (47.2 days ± 36.8 versus 44.7 days ± 52.8, *P* = 0.329). The ALOS of TREATS was shorter (20.5 days ± 20.8) compared against historicals (24.3 days ± 45.9) (*P* = 0.929) and the RLOS of TREATS was longer (26.7 days ± 22.0) compared against historicals (20.4 days ± 16.9) (*P* = 0.089) (Table 5).

The rate of medical complications was 39.7% in the TREATS group and was similar to that of the historical group (37.3%).

The rate of neurosurgical interventions was 35.3% in the TREATS group compared against 43.1% in the historical group.

### 3.3. Regression Analyses

The regression analysis on total FIM gain in TREATS and historicals demonstrated with the intervention of TREATS, there was a statistically significant functional gain of 18.445 points in FIM (95% CI −30.388 to −0.6502) (*P* = 0.03).

The regression model on total FIM gain in the TREATS group estimated about two-thirds of the variance in this variable (adjusted *R* ^2^ = 0.67) (Table 6). Factors associated with a higher FIM gain were shorter RLOS and younger age. Factors associated with a lower FIM gain were higher admission FIM scores. Gender, neurosurgical interventions, and medical complications such as infections, deep venous thrombosis, and seizures were not significantly associated with FIM gain.

## 4. Discussion

Our aims of this study were firstly to prospectively look at the demographics, clinical characteristics of patients of all TBI severities admitted into the acute hospital, and secondly to determine whether early screening and provision of very early integrated TBI rehabilitation service changes functional outcomes in the group who received further inpatient rehabilitation.

There are very few studies that looked at early “reach-in” service of patients with TBI within 72 hours of neurosurgical admission and these studies mainly looked at selected cohorts of patients with TBI especially severe TBI [28–30]. Our study looked at early rehabilitation of patients of all TBI severities. We are not aware of any studies similar to ours locally.

The most common etiology of TBI in our total cohort of 298 screened patients was falls followed by road traffic accidents. This could be due to our cohort that was generally older with an average age of 61.8 years and the elderly are at risk of falls due to age-related physical frailty, immobility, and reduced functional capacity. It seems like our elderly are very susceptible to falls with 91.8% of the geriatric age group sustained a fall leading to TBI. This trend was also seen in the United States with falls attributing to 61% of TBI among adults aged 65 and above [31]. This further emphasizes the need to look into fall prevention strategies and prioritize health-care resources for the elderly as our local population is rapidly ageing. By the year 2030, the number of estimated residents in Singapore aged 65 and above will reach about 20% of the total population [32].

The statistically significant difference in age between the TREATS and historical groups could reflect more comprehensive patient screening and identification processes inherent in the early “reach-in” screening program.

In our study, males are almost twice as likely as females to sustain a TBI and this is consistent with the general TBI cohort in both overseas and local settings [7, 33]. This is in spite of our cohort having more falls than road traffic accidents, but the reasons for more males were predisposed to falls is unclear. This observation is different from that seen in some of the local studies which had shown that females tend to fall more than males [34, 35], but the different observations could be related to different cohorts of patients that were selected from different settings.

Although the LOS of the TREATS and historical groups were not statistically different, the observation of the ALOS of the TREATS group was shorter than that of the historical group and the RLOS of the TREATS group was longer than that of the historical group supported the fact that early “reach-in” screening picked up patients with rehabilitation needs earlier. By provision of early screening and integrated TBI rehabilitation, the TREATS group functionally improved.

Both of the ALOS of our total cohort of 298 screened patients with TBI (19.9 days) as well as ALOS of the TREATS group (20.5 days) were shorter compared with a local study on a selected group of patients undergoing TBI rehabilitation in a dedicated facility (34.9 days) [7]. This may be explained by, firstly, average time from injury to admission to TBI rehabilitation unit was shortened with early screening of all patients with TBI admitted into the neurosurgical unit. Secondly, most of our screened cohort and the TREATS group sustained mild TBI with higher average admission GCS scores (GCS 13.6 and 13.0, resp., versus 8.3) which also reflected most of our patients that were discharged home directly.

Mammi et al. carried out a similar study which involved the early participation of a physiatrist in ICU and neurosurgery rounds 3 times a week with the aim to start comprehensive rehabilitation as soon as possible [28]. In their study, 88 patients with TBI were accepted for inpatient rehabilitation after the early screening during the 4-year study period. Their mean ALOS was 18 days which was similar to that of our TREATS group (20.5 days). However, their mean RLOS was longer than our TREATS group (37.0 days versus 26.7 days). This difference could possibly be explained by their group of patients having more severe injuries as reflected by the lower mean GCS (7.01 versus 13.0). Their demographics were also different from our group. The cause of TBI in their cohort was mostly due to motor vehicle accidents (85%), whereas the cause in our TREATS group was mostly related to falls (80.9%). Their patients were younger (35.12 years versus 66.2 years) than our patients in the TREATS group. Both their study and our study also showed a statistically significant gain in FIM after inpatient rehabilitation.

Our study showed a poor relationship between ALOS and age. Frankel et al. also reported that ALOS did not differ between older (age > 44 years) and younger (age ≤ 44) patients [36]. This indicated that although age was one of the prognostic indicators of TBI recovery [37], there could be other more significant factors that impacted ALOS such as the initial GCS and the total FIM on admission.

## 5. Conclusion

In conclusion, our study provided important country and culture specific epidemiological data on an unselected cohort of patients with TBI in Singapore. It was noted that the leading cause of TBI was falls and our patients were generally elderly. This data may serve as an important reference when planning for a national database and could help prioritize limited health-care resources in our local setting. It emphasizes the need for a comprehensive country wide falls prevention initiative especially in the elderly population.

By providing very early integrated TBI rehabilitation within the acute hospital setting, patients with TBI were seen by the rehabilitation team earlier and those with further rehabilitation needs were sent to the dedicated TBI rehabilitation unit earlier. Our results showed that there are functional improvements in the subset of patients who further received inpatient rehabilitation. Our study has shown that it is feasible to provide such an early integrated TBI rehabilitation service within an acute level I trauma center.

As several overseas studies have shown that early rehabilitation improves functional outcomes [8, 9] during the acute hospitalization which in turn leads to significant reduction in economic costs [38, 39], we have plans to continue this program with the aims to collect more data especially assessment of long-term functional outcomes to further determine the long-term benefits and cost effectiveness of such a very early integrated TBI rehabilitation program.

## Figures and Tables

**Table 1:** Age, injury severity, etiology, and FIM scores in 298 men and women.

	Total (*n* = 298)	Men (*n* = 195)	Women (*n* = 103)	*P* value
Age (years)				
Mean (SD)	61.8 (19.1)	58.8 (19.2)	67.6 (17.5)	<0.001
Range	15–99	15–99	16–97	
Severity categories				
Mild (GCS 13–15)	248 (83.2%)	158 (81.0%)	90 (87.4%)	0.37
Moderate (GCS 9–12)	24 (8.1%)	18 (9.2%)	6 (5.8%)	
Severe (GCS 3–8)	26 (8.7%)	19 (9.8%)	7 (6.8%)	
GCS				
Mean	13.6 (2.8)	13.4 (3.0)	13.9 (2.3)	0.17
Median	15	15	15	
Motor FIM				
Mean (SD)	48.3 (29.7)	49.4 (30.2)	46.1 (28.5)	0.28
Median	52.0	55.0	48.0	
Range	13–91	13–91	13–91	
Cognitive FIM				
Mean (SD)	23.3 (12.2)	23.5 (12.1)	23.1 (12.4)	0.64
Median	30.0	30.0	30.0	
Range	5–35	5–35	5–35	
Total FIM				
Mean (SD)	71.6 (40.0)	72.9 (40.5)	69.2 (39.0)	0.30
Median	76.5	83.0	73.0	
Range	18–126	18–126	18–126	
ALOS (days)				
Mean (SD)	19.9 (28.7)	21.0 (29.7)	17.6 (26.6)	0.25
Median	9.0	10.0	8.0	
Range	1–199	1–199	1–176	
TBI Etiology				
Assault	14 (4.7%)	13 (4.4%)	1 (0.3%)	0.03
Fall	231 (77.5%)	143 (48.0%)	88 (29.5%)	
Road traffic accidents (RTA)	30 (10.1%)	19 (6.4%)	11 (3.7%)	
Sports	7 (2.3%)	6 (2.0%)	1 (0.3%)	
Others	16 (5.4%)	14 (4.7%)	2 (0.7%)	

**Table 2:** Distribution of etiology of TBI according to age groups (years) in 298 patients.

TBI etiology	Age group (years)								
	0–19	20–29	30–39	40–49	50–59	60–69	70–79	80–89	≥90
Assault	0	1	7	1	2	2	1	0	0
Fall	2	9	13	17	31	44	59	46	10
RTA	1	3	5	6	4	7	3	1	0
Sports	2	3	0	2	0	0	0	0	0
Others	0	0	2	4	1	4	4	1	0

Total	5	16	27	30	38	57	67	48	10

**Table 3:** Demographics and clinical variables of patients with TBI (*N* = 298).

Variable	Frequency *n* (%)
Age groups	
<50 years old	78 (26.2%)
50–64 years old	74 (24.8%)
≥65 years old	146 (49.0%)
Race	
Chinese	223 (74.8%)
Malay	25 (8.4%)
Indian	25 (8.4%)
Others	25 (8.4%)
Main radiological findings	
Subdural hematoma (SDH)	108 (36.2%)
Subarachnoid hemorrhage (SAH)	53 (17.7%)
SDH and SAH	40 (13.4%)
Concussion	38 (12.8%)
Contusion	19 (6.4%)
Extradural hematoma (EDH)	18 (6.0%)
Intracerebral hemorrhage (ICH)	22 (7.4%)
Types of neurosurgical interventions	
None	236 (79.2%)
Craniectomy	26 (8.7%)
Burr hole surgery	34 (11.4%)
Burr hole surgery and craniectomy	1 (0.3%)
External ventricular drain insertion	1 (0.3%)
Types of infections	
None	250 (83.9%)
Pneumonia	21 (7.0%)
Urinary tract infections (UTI)	17 (5.7%)
Pneumonia and UTI	7 (2.3%)
Others	3 (1.0%)

**Table 4:** RLA in 298 patients with TBI.

	Total (*n* = 298)	Men (*n* = 195)	Women (*n* = 103)
RLA Level			
(i) No response	12 (4.0%)	9 (3.0%)	3 (1.0%)
(ii) Generalised response	6 (2.0%)	3 (1.0%)	3 (1.0%)
(iii) Localised response	13 (4.4%)	10 (3.4%)	3 (1.0%)
(iv) Confused and agitated	14 (4.7%)	13 (4.4%)	1 (0.3%)
(v) Confused and inappropriate	22 (7.4%)	12 (4.0%)	10 (3.4%)
(vi) Confused and appropriate	38 (12.8%)	29 (9.7%)	9 (3.0%)
(vii) Automatic and appropriate	42 (14.1%)	22 (7.4%)	20 (6.7%)
(viii) Purposeful and appropriate	151 (50.7%)	97 (32.6%)	54 (18.1%)

**Table 5:** Demographics and functional outcomes of TREATS versus historicals.

	TREATS (*n* = 68)	Historicals (*n* = 51)	*P* value
Age (years)			
Mean (SD)	66.2 (17.0)	47.8 (20.1)	<0.001
Range	15–92	14–81	
Total FIM on admission			
Mean (SD)	51.2 (32.4)	59.2 (27.2)	0.064
Median	50.5	60.0	
Range	18–126	18–122	
Total FIM on discharge			
Mean (SD)	80.4 (26.7)*	79.2 (27.8)	0.697
Median	89.0	83.0	
Range	18–126	18–123	
FIM gain			
Mean (SD)	29.3 (34.9)	20.0 (23.1)	0.201
Motor FIM gain			
Mean (SD)	22.9 (25.6)	17.5 (19.4)	0.222
Cognitive FIM gain			
Mean (SD)	6.4 (11.7)	2.5 (5.1)	0.216
ALOS (days)			
Mean (SD)	20.5 (20.8)	24.3 (45.9)	0.929
Median	14.0	14.0	
Range	2–115	0–320	
RLOS (days)			
Mean (SD)	26.7 (22.0)	20.4 (16.9)	0.089
Median	20.5	16.0	
Range	2–118	3–105	
Total LOS (days)			
Mean (SD)	47.2 (36.8)	44.7 (52.8)	0.329
Median	32.0	30.0	
Range	13–199	14–348	
TBI etiology			
Assault	3 (4.4%)	3 (5.9%)	
RTA	5 (7.4%)	17 (33.3%)	
Sports	1 (1.5%)	0 (0%)	
Falls	55 (80.9%)	26 (51.0%)	
Others	4 (5.9%)	4 (7.8%)	
Unknown	0 (0%)	1 (2.0%)	
GCS	13.0	11.1	0.005

*P < 0.001.

**Table 6:** Multiple linear regression analysis model on FIM Gain in TREATS group.

Variable	B*	SE	*B*	*P*
Female	−5.11	6.45	−0.64	0.431
Age	−0.60	0.167	−0.29	0.001
Admission cognitive FIM scores	−0.87	0.39	−2.22	0.031
Admission motor FIM scores	−0.81	0.20	−0.52	<0.001
RLOS	−0.38	0.13	−0.24	0.006
Surgical interventions	−4.68	5.82	−0.07	0.424
Complications	−9.41	6.36	−0.13	0.144

B*: unstandardized coefficient; SE: standard error; *B*: standardized coefficient.

Adjusted *R* ^2^ = 0.67.

## Study Limitations

Our study looked at screening patients with TBI of all severities for early acute rehabilitation. This is a heterogeneous group where functional outcomes are widely different.

Although we attempted to collect data on duration of PTA, much of the data was incomplete so we were not able to report PTA duration in the results.

We were not able to carry out a randomized controlled trial because it did not seem ethical to deny patients with TBI early screening and provision of early integrated TBI rehabilitation. Instead, we carried out subgroup analysis of the TREATS group who received further inpatient rehabilitation in a dedicated TBI rehabilitation unit and compared against the historical group who did not undergo early screening.

We recognized that the TREATS group may not be exactly comparable against the historical group as these 2 groups of patients were heterogeneous with a wide spectrum of injury severities and clinical variables. Nevertheless, it provided valuable information regarding functional outcomes of early screening followed by early provision of TBI rehabilitation during acute hospitalization in patients of all TBI severities.

## References

[1] Baguley I, Slewa-Younan S, Lazarus R, Green A (2000). Long-term mortality trends in patients with traumatic brain injury. *Brain Injury*.

[2] http://www.cdc.gov/TraumaticBrainInjury.

[3] http://www.moh.gov.sg/content/moh_web/home/pressRoom/speeches_d/2013/speech-by-director-of-medical-services–prof-k–satkunanantham–.html.

[4] Lee KK, Seow WT, Ng I (2006). Demographical profiles of adult severe traumatic brain injury patients: implications for healthcare planning. *Singapore Medical Journal*.

[5] Finkelstein E, Corso P, Miller TR (2006). *The Incidence and Economic Burden of Injuries in the United States*.

[6] Coronado VG, McGuire LC, Faul M, Zasler ND, Katz DI, Zafonte RD, Arciniegas DB (2012). Traumatic brain injury epidemiology and public health issues. *Brain Injury Medicine: Principles and Practice*.

[7] Chua KSG, Earnest A, Chiong Y, Kong K-H (2010). Characteristics and correlates of rehabilitation charges during inpatient traumatic brain injury rehabilitation in Singapore. *Journal of Rehabilitation Medicine*.

[8] Cope N, Christensen AL, Uzzell BP (1994). Traumatic brain injury rehabilitation outcome studies in the United States. *Brain Injury & Neuropsychological Rehabilitation: International Perspectives*.

[9] Faul M, Wald MM, Rutland-Brown W, Sullivent EE, Sattin RW (2007). Using a cost-benefit analysis to estimate outcomes of a clinical treatment guideline: testing the Brain Trauma Foundation guidelines for the treatment of severe traumatic brain injury. *The Journal of Trauma*.

[10] Cumming TB, Thrift AG, Collier JM (2011). Very early mobilization after stroke fast-tracks return to walking: further results from the phase II AVERT randomized controlled trial. *Stroke*.

[11] Lomber SG, Eggermont JJ (2006). *Reprogramming the Cerebral Cortex: Plasticity Following Central and Peripheral Lesions*.

[12] Biernaskie J, Chernenko G, Corbett D (2004). Efficacy of rehabilitative experience declines with time after focal ischemic brain injury. *Journal of Neuroscience*.

[13] Lippert-Grüner M, Maegele M, Pokorný J (2007). Early rehabilitation model shows positive effects on neural degeneration and recovery from neuromotor deficits following traumatic brain injury. *Physiological Research*.

[14] Craig LE, Bernhardt J, Langhorne P, Wu O (2010). Early mobilization after stroke: an example of an individual patient data meta-analysis of a complex intervention. *Stroke*.

[15] Tyedin K, Cumming TB, Bernhardt J (2010). Quality of life: an important outcome measure in a trial of very early mobilisation after stroke. *Disability and Rehabilitation*.

[16] https://www.tbindsc.org/.

[17] Murray GD, Teasdale GM, Braakman R (1999). The European Brain Injury Consortium survey of head injuries. *Acta Neurochirurgica*.

[18] Jourdan C, Bayen E, Bosserelle V (2013). Referral to rehabilitation after severe traumatic brain injury: results from the PariS-TBI study. *Neurorehabilitation and Neural Repair*.

[19] Rudehill A, Bellander B-M, Weitzberg E, Bredbacka S, Backheden M, Gordon E (2002). Outcome of traumatic brain injuries in 1,508 patients: Impact of prehospital care. *Journal of Neurotrauma*.

[20] He W, Sengupta M, Velkoff VA (2005). *65+ in the United States: 2005*.

[21] Simon K A breakdown on falls in the elderly. http://www.stacommunications.com/journals/cme/2005/April/PDF/059.pdf.

[22] http://www.cdc.gov/concussion/headsup/pdf/Facts_for_Physicians_booklet-a.pdf.

[23] Granger CV, Hamilton BB, Linacre JM, Heinemann AW, Wright BD (1993). Performance profiles of the functional independence measure. *The American Journal of Physical Medicine and Rehabilitation*.

[24] Shores EA, Marosszeky JE, Sandanam J, Batchelor J (1986). Preliminary validation of a clinical scale for measuring the duration of post-traumatic amnesia. *Medical Journal of Australia*.

[25] Marosszeky NEV, Ryan L, Shores EA (1998). *The PTA Protocol: Guidelines for Using the Westmead Post-Traumatic Amnesia (PTA) Scale*.

[26] Cifu DX, Kreutzer JS, Marwitz JH (1996). Functional outcomes of older adults with traumatic brain injury: a prospective, multicenter analysis. *Archives of Physical Medicine and Rehabilitation*.

[27] Fakhry SM, Trask AL, Waller MA, Watts DD, Chendrasekhar A, Hammond JS (2004). Management of brain-injured patients by an evidence-based medicine protocol improves outcomes and decreases hospital charges. *Journal of Trauma*.

[28] Mammi P, Zaccaria B, Franceschini M (2006). Early rehabilitative treatment in patients with traumatic brain injuries: outcome at one-year follow-up. *Europa Medicophysica*.

[29] Lippert-Grüner M (2010). Early rehabilitation of comatose patients after traumatic brain injury. *Neurologia i Neurochirurgia Polska*.

[30] Andelic N, Bautz-Holter E, Ronning P (2012). Does an early onset and continuous chain of rehabilitation improve the long-term functional outcome of patients with severe traumatic brain injury?. *Journal of Neurotrauma*.

[31] Faul M, Xu L, Wald MM (2010). *Traumatic Brain Injury in the United States: Emergency Department Visits, Hospitalizations, and Deaths*.

[32] http://app.msf.gov.sg/Portals/0/Summary/research/CAI_report.pdf.

[33] Langlois JA, Rutland-Brown W, Thomas KE (2006). *Traumatic Brain Injury in the United States: Emergency Department Visits, Hospitalizations, and Deaths*.

[34] Yeo YYC, Lee SK, Lim CY, Quek LS, Ooi SBS (2009). A review of elderly injuries seen in a Singapore emergency department. *Singapore Medical Journal*.

[35] Chan KM, Pang WS, Ee CH (1997). Epidemiology of falls among the elderly community dwellers in Singapore. *Singapore Medical Journal*.

[36] Frankel JE, Marwitz JH, Cifu DX, Kreutzer JS, Englander J, Rosenthal M (2006). A follow-up study of older adults with traumatic brain injury: taking into account decreasing length of stay. *Archives of Physical Medicine and Rehabilitation*.

[37] Kothari S, Zasler ND, Katz DI, Zafonte RD (2007). Prognosis after severe TBI: a practical, evidence based approach. *Brain Injury Medicine: Principles and Practice*.

[38] Wagner AK, Fabio T, Zafonte RD, Goldberg G, Marion DW, Peitzman AB (2003). Physical medicine and rehabilitation consultation: relationships with acute functional outcome, length of stay, and discharge planning after traumatic brain injury. *The American Journal of Physical Medicine and Rehabilitation*.

[39] Sirois MJ, Lavoie A, Dionne CE (2004). Impact of transfer delays to rehabilitation in patients with severe trauma. *Archives of Physical Medicine and Rehabilitation*.

